# ‘I Doubt Myself and Am Losing Everything I Have since COVID Came’—A Case Study of Mental Health and Coping Strategies among Undocumented Myanmar Migrant Workers in Thailand

**DOI:** 10.3390/ijerph192215022

**Published:** 2022-11-15

**Authors:** Tual Sawn Khai, Muhammad Asaduzzaman

**Affiliations:** 1 Sociology and Social Policy, School of Graduate Studies, Lingnan University, Hong Kong, China; 2 Department of Community Medicine and Global Health, Institute of Health and Society, Faculty of Medicine, University of Oslo, 450 Oslo, Norway; 3 Planetary Health Working Group, Be-Cause Health, Institute of Tropical Medicine, Nationalestraat 155, 2000 Antwerp, Belgium; 4 Planetary Health Alliance, Boston, MA 02115, USA

**Keywords:** migrant health, undocumented migrants, COVID-19, mental health, coping strategy, Myanmar, Thailand, mixed method

## Abstract

Migrant populations have always been vulnerable to a high burden of social exclusion, mental disorders, physical illnesses, and economic crises. The current COVID-19 pandemic has further created a frantic plight among them, particularly for undocumented migrant workers in the global south. We have conducted a mixed method study among the undocumented Myanmar migrant workers (UMMWs) in Thailand to explore how the COVID-19 disruption has impacted their mental health and what coping strategies they have adopted. Following the onset of COVID-19 and the recent coup d’état in Myanmar, our current study is the first attempt to understand the mental health status and predicament of this neglected migrant group. A total of 398 UMMWs were included in the online survey, of which 23 participated in qualitative interviews. The major mental health issues reported by the study participants were depression, generalized anxiety disorder, frustration, stress, and panic disorders, while loss of employment, worries about the pandemic, social stigma, lack of access to healthcare, lockdown, and fear of detention were the predominant contributing factors. In response, we identified two key coping mechanisms: coping at a personal level (listening to music, playing online game, praying, and self-motivation) and coping at a social level (chatting with family and friends and visiting religious institutions). These findings point to the importance of policy and intervention programs aimed at upholding mental health at such humanitarian conditions. Sustainable institutional mental health care support and social integration for the migrant workers, irrespective of their legal status, should be ensured.

## 1. Introduction

In 2020, there were 281 million international migrants, which is equivalent to 3.6% of the global population [1]. Due to their inadequate housing, poor hygienic conditions, and impractical social distance practices, migrants have been one of the most vulnerable populations during the unprecedented COVID-19 pandemic [2,3,4]. The current pandemic has had a detrimental effect on this migrant population’s health and socioeconomic standing [5,6,7]. Particularly, the undocumented or irregular migrant workers in the global south are in grave situations due to difficulties in accessing healthcare and social supports [8].

Thailand has emerged as the top destination for economic migrant workers from the Association of Southeast Asian Nations (ASEAN) member countries. Typically, they are employed in 3D jobs (dirty, dangerous, and demanding) which the local people do not want to do, such as construction, agricultural and animal husbandry, services, and fishery-related sectors (seafood) [9]. As of 2019, approximately 4.9 million foreign workers live in Thailand, and migrant workers from Myanmar account for 68% of this population [10]. However, at least 1.25 million of them are undocumented Myanmar migrant workers (UMMWs) who are not covered by the social security schemes (SSS) [11]. Despite the COVID-19 spike in 2020, which resulted in the return of 60,000 to 2,000,000 migrant workers to their home countries from Thailand, Myanmar migrant workers were unable to do so due to the closing of the border, the military coup, and political unrest in their home country [12].Thus, the number of UMMWs is expected to rise after the COVID-19 disruption due to difficulties in extending work permits or passports as a result of office closures, community lockdowns, and travel restrictions.. For example, in December 2020, Thai authorities had locked down approximately 40,000 Myanmar migrant workers at their residences in the Samut Sakhon province to avoid the transmission of COVID-19 infection [13]. Unfortunately, Thailand’s government does not have housing legislation for migrant workers. Migrant workers are, therefore, exposed to higher risks for contracting COVID-19 due to low convergence rates of masking, overcrowded housing conditions, and impractical social distancing. Up to April 2021, approximately 166,000 COVID-19 infections were recorded among migrant workers, which was 7.8% of the total infections in Thailand at that time [14]. In addition, the migrant workers also suffered from various mental health issues which are not documented.

Many of the migrant workers who had COVID-19 were laid-off by their employers because of concerns that they may spread the disease. They have also been evicted from their homes because they were living in accommodations provided by their employers and were unable to pay their rent [15]. An estimated 2 million undocumented migrants are excluded from the nationwide COVID-19 vaccination program and are ineligible for government monetary support [16]. They have limited access to COVID-19 testing and medical treatment due to cost concerns and fears of being imprisoned and deported [15]. They are burdened with debt and no source of income, which adds to their psychological stress [17].

The aims of this study were to explore how the COVID-19 pandemic has impacted the mental health of UMMWs in Thailand and what coping strategies they have adopted. Understanding their hardships and problems is critical for providing the comprehensive support needed since healthy workers contribute to the economic success of both their home country and their destination country.

## 2. Materials and Methods

### 2.1. Study Settings and Study Design

This is a convergent parallel mixed method study among UMMWs in Thailand. This study was conducted between 1 September 2021–31 January 2022. Since the enforcement of travel restrictions and the surge of COVID-19 infections occurred during the period of data collection, both the survey and in-depth interviews were conducted online. The participants in this study were from different districts in Thailand. A convergent parallel mixed method research design combined the qualitative and quantitative data collection and interpretation to answer a single (or a set) of research questions [18].

### 2.2. Data Collection Procedure

We employed multistage snowball sampling to find the participants. First, online survey questionnaires were distributed to a number of migrant worker NGOs and community-based organizations, migrant self-help organizations, and migrant communities via online platforms (Messenger and WhatsApp—both are subsidiaries of Facebook, Inc., Menlo Park, CA, USA) group chats in Thailand. The questionnaires were adapted from an earlier survey conducted by the International Organization for Migration [19] and Baloran et al. [20] to fit the objectives of this study. A total of 398 UMMWs participated in the online survey.

At the end of the online survey, the participants were asked to provide their contact details if they were willing to participate in the further qualitative study. When we contacted the willing participants by phone and social media, the details of the study were explained to them and participants were recruited for in-depth interviews (IDIs) or focus group ddiscussions (FGDs), depending on their availability. Before conducting the interviews/FGDs, the study objectives, as well as the interview protocol, were explained, which included their voluntary participation and the freedom to withdraw at any point during the interview. The interviews were conducted in Myanmar language using the participants’ preferred internet platforms either Zoom (Zoom Video Communications, Inc., San Jose, CA, USA) or WhatsApp, which enabled end-to-end encryption to protect their personal information. The researcher took notes during the interview and recorded the entire audio conversation.

Participants were also invited to recommend or recruit more members who met the interview requirements. All of the participants were asked about how the COVID-19 pandemic affected their livelihood and mental health, and how they coped to overcome those challenges.

This study included 23 UMMWs who had been working and residing in Thailand for at least one year prior to the start of the COVID-19 pandemic. Among the participants, 11 were interviewed using semi-structured questions, while the other 12 were interviewed employing FDGs. There were 11 men and 12 women among the 23 participants. Among them, 15 were unemployed due to the COVID-19 disruption and only 6 of them had employment at the time of the interviews. However, the majority of the employed participants were self-employed or worked on a part-time basis. The youngest participant’s age was 19 and the oldest was 65. Moreover, 6 participants were infected with COVID-19 at the time of interview.

### 2.3. Ethical Consideration

Before beginning the research, ethical approval was obtained from the Postgraduate Student Committee (PSC) of Lingnan University in Hong Kong. Throughout the data collection procedure, the researchers adhered to study ethics, and oral consent was obtained before conducting interviews and audio recordings. The written consent was inconvenient for the migrant workers since they had difficulties accessing computers, printing, or scanning after signature. The researchers also informed them that their personal profile would be kept strictly confidential and would be utilized under a pseudonym.

### 2.4. Data Analysis

The quantitative data analysis was performed using IBM SPSS Statistic for Windows, Version 26 (IBM, Armonk, NY, USA). To examine how COVID-19 had affected the mental health of the UMMWs in Thailand and how they coped, we used descriptive statistics, rather than regression statistical tests, to determine the factors that had most impacted them or whether their gender or social networking played an influential role in their mental health and ability to cope. The quantitative data are presented using percentages and frequencies to describe sociodemographic characteristics, mental health status, mental health symptom experiences, and coping mechanisms. For example, for mental health status, the online survey participants were asked (yes or no) ‘whether you believed your mental health had deteriorated as a result of the pandemic’.

Moreover, in order to fully understand and familiarize ourselves with the qualitative data, we replayed the audio recordings several times before transcribing. A direct transcription of the audio recording in the English language was then conducted verbatim using an interpretive approach that focused on the participant’s perceptions and an interpretation of the discussion. To ensure quality control, the entire audio recording was listened to multiple times and the English transcription was verified by reading it line by line. It was then matched with the field notes to avoid errors or biases in the transcription. Next, we utilized the thematic analysis guidelines developed by Braun et al. [21] to identify the various themes that emerged from the interviews and categorized them into several subtopics based on the similar experiences shared by the respondents.

## 3. Results

### 3.1. Sociodemographic Characteristics of the Survey Participants

Table 1 provides the brief sociodemographic characteristics of the survey participants.

Table 2 provides the brief sociodemographic characteristics of the interview participants.

### 3.2. Migrant Workers’ Mental Health Condition (from Quantitative Survey)

The mental health status of Myanmar migrant workers in Thailand has deteriorated following the COVID-19 pandemic, as reported by more than half (61.8%) of the 398 online survey participants.

**Figure 1:**
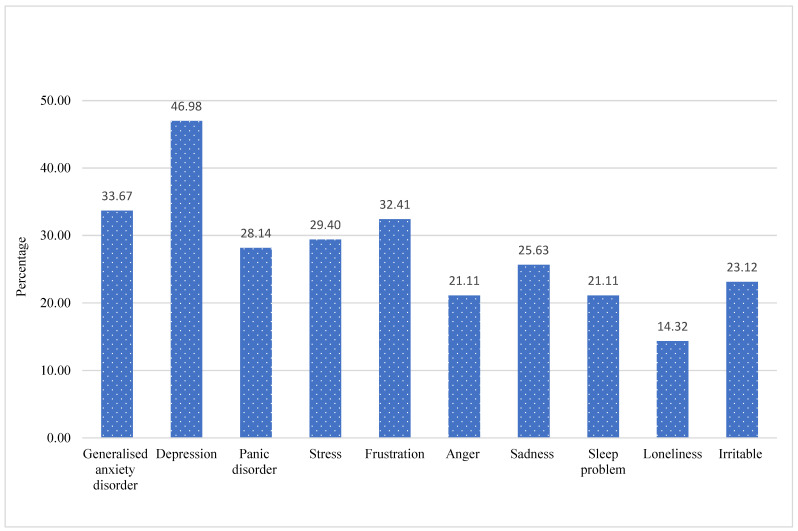
Mental health symptom experiences.

The top five mental health issues reported by the study participants were depression (46.98%), generalized anxiety disorder (33.67%), frustration (32.41%), stress (29.40%), and panic disorder (28.14%), as illustrated in Figure 1.

### 3.3. Major Factors That Influenced Mental Health

#### 3.3.1. Loss of Employment in COVID-19

The survey indicated depression as the most prevalent mental health problem among Myanmar migrant workers because of the COVID-19 disruption and loss of employment (Figure 1). Many of these migrant workers had large families in their home countries who relied on the workers’ remittances to maintain their everyday lives. On the other hand, since the COVID-19 surge, many of them had lost their jobs and were struggling to survive, owing to a lack of support and an inability to send money to their loved ones.

*‘There are no jobs available during COVID-19. Lack of employment is a significant issue since there was no income to survive. I was seriously depressed because I didn’t have the income to feed my children due to job loss’*

—P9 (F).

Furthermore, nearly half (42.2%) of the survey respondents believed that the COVID-19 pandemic had made them hopeless. Similarly, several participants claimed that they had considered suicide since they had no income to support for their family members.

*‘My mental health deteriorated for around 5 months during the early phases of COVID-19 in 2020. I am disappointed with my future and have lost my interest in living’*

—P6 (M).

*‘I have suffered from depression due to a lack of regular work and income throughout this COVID-19’*

—P1 (M).

#### 3.3.2. Worries about Susceptibility to COVID-19

Most migrant workers stayed in the employer-provided housing with their coworkers or family members to reduce their living expenses. Their rooms were tiny, and they were in precarious settings where social distancing was difficult, making it hard to avoid COVID-19 infection. Participants stated that a large number of migrant patients were sent to a quarantine facility which was not equipped with proper healthcare services, and many of them took care of themselves by taking pills such as paracetamol. Therefore, they were especially worried when someone near their community was infected.

*‘I doubt myself and am losing everything I have since COVID came. Because the virus may dwell in money, walls, and other places. Although I usually wore a mask and washed my hands frequently, I felt insecure, which caused me mental fatigue. I felt more mentally stressed when the positive cases were confirmed near the community’*

—FGD1 participant.

The migrant workers’ mental health had deteriorated due to concerns of losing their jobs if they tested positive for COVID-19 since many of their coworkers who were infected had been fired by their employers without any compensation. Such conditions made it difficult to sleep and caused overthinking.

*‘My mental health was suffering the consequences of my anxiety of losing my job and income if I was infected with COVID-19’*

—FDG 2 participant.

*‘There were times when I couldn’t sleep at all. I feel depressed and anxious about contracting with the COVID-19’*

—P10 (F).

#### 3.3.3. Increased Infection Risk with COVID-19 and Denied Access to Healthcare

The entire family or residence was at risk of contacting COVID-19 if one of the individuals became infected due to overcrowding living conditions. Some participants had been infected with COVID-19 and had shared that their mental health deteriorated as they were in foreign countries.

*‘When I tested positive for COVID-19, I experienced extreme depression because I lost my job; my entire family also tested positive, making it difficult to sleep and eat’*

—P5 (M).

Furthermore, migrant workers were denied access to medical treatment from public—and even private—healthcare facilities due to certain allegations that portrayed migrant workers as having spread the virus. Such public discrimination and denial of access to healthcare services had a significantly negative impact on the migrant worker’s mental health.

*‘While my whole family members were diagnosed with COVID-19, we were unable to get medical treatment in a hospital because Myanmar migrant workers were rejected. I was terrified, depressed, and anxious about what might happen and how I would feel. There had been a lot of sadness, since we are not in our home country’*

—FDG 2 participant.

*‘We take several medications on our own during the COVID-19 infection. Due to a lack of access to appropriate medical treatment, I suffered from insomnia, mental depression, and irritation’*

—FDG 2 participant.

Specifically, the participants mentioned that migrant workers who had no social networks and who worked in remote areas may have experienced serious mental health issues as a consequence of loss of employment, lack of access to medical treatment, and lack of mental and social supports.

#### 3.3.4. Lockdown and Daily Living Crisis

Many migrant workers were caught in community lockdowns and could not go anywhere. They could not go to work to earn incomes for basic living expenses. Moreover, they received no support from the Thai government or Myanmar’s official representatives in Thailand. As a result, they faced starvation and mental depression.

*‘I was also frustrated by the travel restrictions that prohibited going anywhere including to work. In my mind, there are times when I wanted to take my life. I was unable to sleep for a month and was felt like insane’*

—P9 (F).

*‘There are days when there was nothing to eat because I have no money and receive no assistance’*

—P7 (M).

#### 3.3.5. Worries about Family Members’ Safety from COVID-19 Infection

Following the military takeover on 1 February 2021, Myanmar’s health system had depreciated to being on the brink of collapse, and COVID-19 infection cases had increased significantly during the third wave. As a consequence, migrant workers’ mental health became worse as they worried about the safety of their family members.

*‘My family is in Myanmar, and I am in Thailand. My mental health has deteriorated dramatically since I worried whether family members at home can handle or protect from COVID-19 infection’*

—FGD1 participant.

In particular, the mental health conditions appeared to have worsened among individuals who were single children who had left their elderly parents, as they were concerned that the elderly were more likely to become infected and had higher mortality rates around the globe.

*‘In Thailand, I am alone, and my mother is also at home alone. As a consequence, I am concerned for my mother during COVID-19. I sometimes wonder if I have a wing to return home. Sometimes I wish I could go somewhere where no one could hear me scream and cry. I am missing my mother’*

—FGD1 participant.

#### 3.3.6. Fear of Detention

Migrant workers were not only afraid of being infected by COVID-19, they were also afraid of imprisonment since Thai authorities had been conducting random raids in the name of COVID-19 prevention measures and were placing those detained into detention centers where social distancing was impractical.

*‘I am worried and afraid of the police rather than contacting COVID-19. I am frightened, and I feel like crazy when I think. Now, the police raid homes and arrest irregular migrant workers, and I cannot sleep because I am afraid of being arrested’*

—FGD1 participant.

#### 3.3.7. Fear of Next Waves

The majority of migrant workers had lost their jobs and were facing a humanitarian crisis following the outbreak of the COVID-19 epidemic. They were already traumatized and concerned about the next wave if community lockdowns were enforced since many of their family members relied on their remittances for living expenses.

*‘I have been out of work since the COVID-19 outbreak. I am concerned about the next wave if another lockdown would be imposed’*

—FGD1 participant.

### 3.4. Coping Strategies of UMMWs for Mental Health Adaptation

The mental health coping strategies among UMMWs were diverse, as illustrated in Table 3.

#### 3.4.1. Coping Strategies to Avoid Infection and Mental Distress

To cope with COVID-19 infection and anxiety, most (84.2%) of our survey respondents followed strict personal protective measures (e.g., masking, handwashing, etc.) while 74.9% avoided going out in public places to minimize exposure to COVID-19.

*‘I avoid going to crowded or public places by wearing a mask and washing my hands’*

—FGD2 participant.

#### 3.4.2. Using Social Media Platforms

Almost half (37.7%) of the survey respondents utilized social media to cope with their psychological anguish and loneliness a result of the COVID-19 disruption. Notably, several migrant workers reported feeling encouraged and psychologically strong after listening to some speakers’ social media posts and sharing their stories.

*‘I was motivated by some motivational Facebook posts. Despite the fact that I did not receive encouragement from others, I improved my mental health by listening to them’*

—P6 (M).

#### 3.4.3. Chatting with Family Members and Friends

According to one-third (31.9%) of the survey respondents, chatting with family and friends to relieve stress and obtain support was the most common practice among migrant workers to cope with the consequences of the COVID-19 disruption.

*‘I was able to develop my mental health with the encouragement and inspiration from others since I had heard that some individuals were in worse condition than I was’*

—P8 (F).

On the other hand, sharing psychological counselling and motivation among COVID-19 patients at migrant workers’ quarantine facilities helped them to overcome loneliness and depression, as reported by some participants who had been infected.

*‘There were many people at the quarantine center, and we were motivated to be strong and support one another’*

—FGD1 participant.

#### 3.4.4. Religious Coping

Praying (including reading bibles and practicing worship) was also a common practice among migrant workers for overcoming anxiety and sadness.

*‘I pray every night to overcome depression caused by COVID-19. I occasionally sang a religious song to heal my worries’*

—P10 (F).

*‘I might be able to protect myself. But I am still concerned about whether my family will be secure or infected with COVID-19. I cope with all my trouble through praying to God’*

—FGD2 participant.

Several participants thanked their God for hearing their prayers and sending different donors to provide them with food as they faced darkness, fears, and helplessness.

*‘I suffered moments of sadness and felt hopeless about my life in many ways. I cannot find employment in Thailand because of the COVID-19. I do not intend to return to Myanmar because the situation worsens following the military coup. Sometimes I felt utterly helpless about how to survive, what to eat, and where to live. So, I spend the entire day praying to God. God has heard my prayer, and I can still manage from various donations for food and shelter’*

—P3 (M).

Additionally, several migrant workers reported that chatting with religious leaders and participating in group prayers on social media helped them to overcome stress and sadness.

*‘I pray to God to take away my worries. Since I cannot attend church in person due to social gathering restrictions, I call the pastor and request them to pray for me. We motivate one another and chat about God. As a reason, I do not find it so challenging to handle COVID-19, and I believe it will be over soon’*

—FGD1 participant.

Some migrant workers stated that they eventually considered the COVID-19 pandemic as a part of their life’s journey experiences and managed to cope by praying that it would disappear soon.

*‘This is the nature of life: something will arrive, and something will depart. By taking care of my health, I can only pray that this pandemic will be ended soo’*

—FGD1 participant.

#### 3.4.5. Self-Motivation

Self-motivation was mentioned by migrant workers as one of the tools they utilized to cope with their mental suffering and unemployment challenges following the COVID-19 interruption. One-fifth (21.6%) of the survey respondents motivated themselves to face the COVID-19 outbreak with a positive thought that a better life was still on the horizon and that the pandemic would be curbed someday.

*‘I am not alone facing this pandemic. Other people too. Such thinking allows to overcome my mental health’*

—FGD2 participant.

*‘Because of COVID-19, I experienced depression, frustration, and insomnia. However, I realized that this pandemic affects the entire world, not just myself or my country. If I cannot resist, how can other people too? I am motivating myself to endure this epidemic’*

—FGD2 participant.

*‘As a mother, I keep myself from sinking into depression. We all have a future, and my children do as well. Time will cure this epidemic. So, I try to overcome as much as I can’*

—P2 (F).

Some migrant workers emphasized the need to remain psychologically strong and motivated following the pandemic crisis for the sake of their family and social well-being since no one would look after their family members if something unfortunate happened to them.

*‘Although I was depressed and wanted to commit suicide, I encouraged myself to be strong since I have children, and they must live. I convinced myself that I could do it and not give up no matter what condition’*

—P9 (F).

*‘I was depressed, but not so much to end my life condition. I encouraged myself to survive by telling myself that no one would feed my family if I could not work or survive’*

—FGD2 participant.

#### 3.4.6. Listening to Music and Playing Mobile Game

Over one-fourth (25.1%) of the migrant workers indicated that they listened to music to cope with their anxiety.

*‘I listen to music to cope with my mental problem. I also pray to be free soon from COVID-19’*

—P7 (M).

Some migrant workers (12.8%) reported playing mobile games to improve their mental health since they focused less on their worries and the COVID-19 situation.

*‘Compared to the early phases of COVID-19, my mental health is improving now. I listen to music, play mobile games, and watch movies to get rid of my depression although I could not go out and interact with my friends’*

—P6 (M).

## 4. Discussion

In this research, we aimed to study how the COVID-19 pandemic affected the mental health and well-being of the UMMWs in Thailand, along with studying their coping strategies in response to their mental health conditions. This study is novel and exploratory for several reasons. Thus far, to our knowledge, the mental health issues among undocumented Myanmar migrants in Thailand has never been explored. Secondly, the study population found mental health issues and COVID-19 to be interconnected, and therefore, their coping strategies for both mental health issues and infection are described herein. Finally, this is the first study on this population after the pandemic and the military coup in Myanmar. The migrant workers from Myanmar in Thailand are a vulnerable population in terms of mental health issues. They have reported high mental stress due to language barriers, discernment, and tough working situations and living conditions [22].

Depression and anxiety are the most common mental health issues among migrant workers worldwide [23,24,25,26,27]. Globally, depression and anxiety are present among 38.99% (95% CI = 0.27, 0.51) and 27.31% (95% CI = 0.06, 0.58) of migrant workers, respectively [28]. This statistic is in alignment with our study’s findings. Depression (47%) was the major mental health problem in our study, followed by anxiety disorder (34%). The high prevalence (70.8%) of depression among similar populations (Myanmar migrant workers) in Malaysia [29] supports this finding, and most of them (79.2%) had poor mental well-being, according to the WHO-5 Well-Being Index Scale. Another study conducted in North India shows almost similar depression rates (73%) among migrant workers amidst COVID-19 [30]. However, in another study among Myanmar migrant workers in Thailand (similar to ours), only 11.9% had suffered from the symptoms of depression and/or anxiety before the COVID-19 pandemic [31], but this study did not mention undocumented migrants as a study population and it was conducted before the COVID-19 pandemic. Our research clearly depicts the detrimental effects of the pandemic on the mental well-being, particularly the depressive and anxiety symptoms, of our study population. In addition, the other mental health issues (such as frustration, stress, sleeping disorders, and panic disorder) observed in our study participants are also common in migrant workers [27,28], and COVID-19 was a triggering factor that escalated them.

From the qualitative investigation, we explored the causative factors behind these mental health problems. Loss of employment during the COVID-19 pandemic and worries about being infected with COVID-19 were the major concerns for the migrant workers. Particularly, unemployment during the COVID-19 pandemic was a great threat to the mental well-being and livelihood of the migrant workers, especially those with temporary employment status. The sudden loss of a job resulted in poverty, lack of food, and failure to maintain one’s family in their home country. Fears of infection for themselves and their family members and lockdowns at larger scales made the situation worse, while jobless workers could not even search for jobs and earn incomes for daily expenditures. All these phenomena raised the depression, anxiety, and suicidal tendencies among Myanmar migrant workers in Thailand. Such situations were also observed in similar settings, such as India [32,33]. Inadequate or denial of access to healthcare services for migrants after contacting COVID is a global truth, irrespective of location [34,35,36]. Our study participants also had bitter experiences, having been denied access to both public and private healthcare systems, in addition to the false public and institutional allegations of migrants spreading COVID. Inadequate/denial of access to treatment and healthcare facilities further affected the migrant workers and made them feel socially unacceptable. A global survey on migrants’ mental health found such stressors (unmet basic, medical, and social needs) and discrimination significantly associated with mental problems, especially depression and anxiety [37].

While most of the contributing factors to the mental health issues among Myanmar migrants in Thailand shared a similar global trajectory, we found an unusual and terrifying issue responsible for their mental disharmony, which they labeled as ‘Fear of detention’. The Thai authorities were conducting numerous raids in the name of COVID-19 prevention measures and placing those detained into detention centers where social distancing was impractical, and the undocumented migrant workers were being detained for uncertain durations [38,39]. The avoidance of healthcare stations by undocumented migrants was witnessed in many countries, including developed ones such as the UK, due to the data sharing policy for COVID patients within home ministries [40]. When the global authorities were trying to ensure the equitable distribution of COVID treatments and vaccinations, such initiatives increased the mistrust and delayed control associated with this deadly pandemic. The International Organization for Migration (IOM) has also identified the fear of arrest or deportation and mistrust as major barriers to the migrants’ access to COVID-19 vaccination [41], and this applies to migrants’ healthcare-seeking behaviors, as well.

To manage their acculturative stress, migrant workers always followed some coping strategies that were usually dependent on their socioeconomic resources, acculturative stressors, migration status in their host country, and cultural background/attachment [42,43,44,45]. Though the current pandemic was the added stressor for them, the coping mechanisms were mostly similar. We have classified their coping strategies into two levels: personal and social.

### 4.1. Coping at a Personal Level

Many participants reported trying affordable relaxation activities such as listening to music or playing online games. There is a considerable amount of evidences which depicts the positive impacts of art work and listening to music on mental well-being [46]. However, online game engagement has controversial effects on mental health [47,48]. Interestingly, the Myanmar migrant workers had perceived infection prevention (IP) as one of the major strategies for avoiding stress. Despite economic and social hardship and reduced access to healthcare, most of them (75–84%) strictly maintained personal protective measures such as the use of masks, handwashing, and avoiding public gathering places to minimize exposure to COVID-19. However, this behavior can be explained by the protection motivation theory [49], a famous theory in the health promotion and disease prevention field proposed by Ronald W. Rogers. In this theory, the ‘coping appraisal’ follows the ‘threat appraisal’ when people perceive a certain health issue as an alarming threat and, at the same time, believe in several containment steps to halt the disease process and stress [50]. This theory is applicable to ‘any threat for which there is an effective recommended response that can be carried out by the individual’ [51]. Without the effective community engagement of this vulnerable group, the high uptake of IP measures is encouraging and clearly shows its possibility as a mental health support initiative in the future.

### 4.2. Coping at a Social Level

Migrant workers always face a lack of mental health supports, but undocumented migrant workers also have a high burden of stress. Having no strong bonding in their local community, they try to be in touch with their distant, but own, community in their home country. Almost one-third of the participants found family and friends to be a source of relaxation from stress, such as passing time or chatting with family and friends (through the Internet). This finding is supported by previous studies [52,53]. Usually, people with no or limited support from employers and authorities during the COVID-19 pandemic have shown this tendency [54,55]. In fact, humans are born with a natural ability to recuperate from adversity, which is called resilience. This current scenario is likely a great example of ‘Community Resilience’, which Magis described as a strong social sustainability theory and defined as the ‘’existence, development and engagement of community resources by community members to thrive in an environment characterized by change, uncertainty, unpredictability and surprise.’’ [56]. Such a social resilience approach is practiced more for coping due to its high strength-based focus on an issue instead of a problem or a deficit of focus [57], and community members feel safe and strong enough to face any adversity together.

It has been discussed that feeling isolated or depressed or having insufficient resources and time for self-reflection has always been linked with faith and religion dependency for migrant workers as a coping step [58,59,60]. Practicing religious rituals is more often tied to social assimilation in groups with the same interest, and this is also evident in our study. Approximately one-third of our participants relieved their stress through reading a bible and worshiping a god, chatting with religious leaders, and participating in group prayers. Dependence on faith and religion in stressful situations is very common among migrant workers from Southeast Asia and Africa, and these findings are in line with other studies [42,60,61,62]. Such religious or spiritual coping is also a very common discourse in psychosocial theories, which are mostly described either as a ‘Cognitive Schema’ or ‘Transactional Model’. In a cognitive model, with a religious engagement or spiritual belief, one can be elude an adverse situation which is under the control of a supreme power, and thus be optimistic about tackling stress [63]. According to the transactional model, a person’s cognitive assessment of a circumstance and their choice of coping mechanisms can moderate the influence of life event stress [59]. In this model, the coping strategies can be individual (e.g., reading a bible) and public (e.g., group prayer), which we observed among our study participants.

Again, workers with lower wages, higher numbers of family dependents in their home country, and lesser knowledge about the level of health system or insurance policies are supposed to suffer more from mental stress, with lower numbers of healthcare visits [64]. Undoubtedly, the situation is worse for undocumented migrant workers. Only 4 out of 398 survey participants in our study (less than 1%) sought professional support for their mental health situation. This depicts the lack of general awareness about the mental well-being and insufficient mental-health-seeking behaviors in this group. However, amidst enormous suffering, many migrant workers showed more coping capacity than others to face mental stress, which is explained by Antonovsky as the theory of the sense of coherence (SOC) [65].

### 4.3. Limitations

Though our study provides important insights into migrant mental health, we have limitations. Firstly, we had to conduct an online survey and interview due to the COVID situation. A face-to-face interview would yield more detailed information. Secondly, our study included only 23 participants for the qualitative interview. However, we have been able to capture the major mental health and coping issues from this gender-equal (male = 11, female = 12) sample population. Most importantly, a majority of the interviewed participants (15 out of 23) lost their jobs due to the pandemic and one-fourth (6 out of 23) were infected at the time of the interview, which provided a real-time experience of the research question.

## 5. Practical Policy Implication

The current study identified various factors that adversely impacted the mental well-being of UMMWs in Thailand, such as loss of employment, precarious living conditions, exclusion or denial of healthcare, fear of detention, and community lockdown. All these undermining factors might be relevant to the mental health issues of migrants in a majority of the countries in the global south and developed countries, to some extent. In this context, our study has some crucial policy implications. Firstly, migrant workers should be included in humanitarian response efforts, such as the distribution of basic groceries and basic medical supplies, to support their survivors’ journeys.

Secondly, many migrant workers have been terminated from their employment in Thailand without being compensated. This has significantly affected their mental health, whether directly or indirectly, as they fear another wave and being fired if they become infected with COVID-19. Therefore, it is imperative that the Thai government take concrete actions to prevent such events from occurring and assist those that have been affected through receiving compensation.

Thirdly, this study observed poor medical care and treatment in isolated facilities for those infected with COVID-19, resulting in mental illness and depression in these patients. It is a fundamental human right to have access to health care. The government needs to ensure that everyone in Thailand has fair access to health care, regardless of their immigration status.

Lastly, the Thai government has taken advantage of the movement control enacted previously to curb COVID-19 by raiding irregular migrants and arresting a number of them. We found that some participants were more concerned about the possibility of being detained than they were about being infected with COVID-19. In light of the global health crisis, the Thai government should grant amnesty to all undocumented migrant workers instead of arresting them and requiring them to be registered. As undocumented migrant workers, they lose employment and struggle for family survival, rather than obtaining registration, which is costly.

## 6. Conclusions

The migrant workers of Myanmar in Thailand are undoubtedly one of the most vulnerable populations in the global south due to political turmoil in their home country, workplace stressors, social isolation, undocumented migration status, and the current effects of the COVID-19 pandemic. Unfortunately, most of the challenges they are confronting seem to persist. Unemployed and undocumented persons tend to suffer from long and worsening mental health issues in uncertain situations such as the COVID-19 pandemic. Our current study has not only identified the major mental health challenges, but also the most-practiced coping strategies of this migrant population. These findings can be used as the guiding principles for further longitudinal/follow-up studies, as well as for adopting prevention and intervention programs at both the individual and social level. We strongly argue for incorporating effective health policies in Thailand and any other similar settings to ensure social and institutional mental health and healthcare supports for migrant workers, irrespective of their legal status.

## Figures and Tables

**Table 1:** Sociodemographic characteristics of the survey participants (n = 398).

Sample Characteristic	Frequency	Percentage
Gender		
Male	188	47.2
Female	210	52.8
Age		
18–29	139	34.9
30–39	145	36.4
40–49	80	20.1
50 and above	34	8.5
Current employment status		
Unemployed	156	39.2
Employed	242	60.8
Current employment sector (top 5 answers only)		
Construction	56	23.1
Agriculture sector	49	20.2
Domestic work	34	14.0
Factory/garment production	26	10.7
Retail trade and vendor	14	4.8

**Table 2:** Sociodemographic characteristics of the qualitative study participants (IDIs and FGDs) (n = 23).

List of Participants	Gender	Age	Education Background	Religion	COVID-19 Infection	Employment Status during Interview	The Last and Current Employment Sector	Years of Stay in Thailand
In depth interview (n = 11)								
P1	M	22	Primary	Buddhist	No	Unemployed	Seafood processing	2
P2	F	33	Bachelor	Buddhist	No	Unemployed	Teacher in migrant school	16
P3	M	26	High school	Christian	No	Unemployed	Garment factory	11
P4	F	46	Monastic	Muslim	No	Unemployed	Domestic work	17
P5	F	29	Secondary	Buddhist	Yes	Unemployed	Daily basis	13
P6	M	22	Secondary	Buddhist	No	Part time	Restaurant	12
P7	M	22	Primary	Buddhist	No	Part time	Construction	8
P8	F	40	High school	Buddhist	No	Unemployed	Vendor/self employed	5
P9	F	25	High school	Buddhist	No	Unemployed	Vendor/self employed	6
P10	F	37	Secondary	Buddhist	No	Unemployed	Factory	20
P11	M	21	Primary	Buddhist	No	Part time	Construction	10
Focus group discussion (n = 2)								
FGD-1	F	65	High school	Christian	Yes	Unemployed	Self-business	10
FGD-1	F	29	High school	Buddhist	No	Unemployed	Beauty parlor	6
FGD-1	M	50	Secondary	Buddhist	No	Employed	Agriculture sector/farm	27
FGD-1	F	35	Monastic	Christian	No	Unemployed	Cleaner at public school	9
FGD-1	M	27	Secondary	Christian	Yes	Unemployed	Garment factory	9
FGD-1	M	28	High school	Buddhist	No	Unemployed	Daily basis	14
FGD-2	F	24	High school	Buddhist	No	Unemployed	Cleaner at hotel	5
FGD-2	M	44	Secondary	Buddhist	Yes	Employed	Multiple jobs/care workshop	9
FGD-2	F	38	High school	Buddhist	Yes	Self-business	Convenience store	15
FGD-2	M	54	Bachelor	Buddhist	Yes	Legal advisor	Legal advisor	13
FGD-2	M	19	High school	Buddhist	No	Unemployed	Factory	3
FGD-2	F	19	High school	Buddhist	No	Employed	Factory	10

**Table 3:** Coping strategies for COVID-19 infection and mental health.

Coping Strategies	Percentage	Frequency
A. Infection prevention measures		
Follow strict personal protective measures (e.g., masking, handwashing, etc.)	84.2	335
Avoid going out in public places to minimize exposure to COVID-19	74.9	298
B. Relaxation activities		
Do relaxing activities, such as listening to music	25.1	100
Play online games	12.8	51
Avoid media news about COVID-19 and related fatalities	6.5	26
Vent emotions by crying, screaming, etc.	5.3	21
C. Religious coping		
Praying, worshipping, reading bible, etc.	29.1	116
D. Using social media		
Use social media such as Facebook	37.7	150
E. Family support		
Chat with family members and friends to relieve stress and obtain support	31.9	127
F. Self-motivation		
Talk to and motivate myself to face the COVID-19 outbreak with a positive outlook	21.6	86
Try to be busy at work to keep your mind off of COVID-19	7.0	28
G. Professional support		
Consult doctors to reduce stress	1.0	4

## Data Availability

All data are available in the manuscript.

## References

[1] Nations U. (2021). International Migrant Stock 2020. https://www.un.org/development/desa/pd/content/international-migrant-stock.

[2] Lancet T. (2020). India under COVID-19 lockdown. Lancet.

[3] ECDC (2021). Reducing COVID-19 Transmission and Strengthening Vaccine Uptake among Migrant Populations in the EU/EEA.

[4] Moroz H., Shrestha M., Testaverde M. (2020). Potential Responses to the COVID-19 Outbreak in Support of Migrant Workers.

[5] Lewis N.M., Friedrichs M., Wagstaff S., Sage K., LaCross N., Bui D., McCaffrey K., Barbeau B., George A., Rose C. (2020). Disparities in COVID-19 incidence, hospitalizations, and testing, by area-level deprivation—Utah, March 3–July 9, 2020. Morb. Mortal. Wkly. Rep..

[6] Liem A., Wang C., Wariyanti Y., Latkin C.A., Hall B.J. (2020). The neglected health of international migrant workers in the COVID-19 epidemic. Lancet Psychiatry.

[7] News U. (2022). WHO Calls for Action to Provide Migrant and Refugee Healthcare. https://news.un.org/en/story/2022/07/1122872.

[8] World Health Organization (WHO) (2021). COVID-19 Immunization in Refugees and Migrants: Principles and Key Considerations: Interim Guidance.

[9] Chantavanich S., Vungsiriphisal P. (2012). Myanmar migrants to Thailand: Economic analysis and implications to Myanmar development. Economic Reforms in Myanmar: Pathways and Prospects.

[10] United Nations Thematic Working Group on Migration in Thailand Thailand Migration Report 2019. https://thailand.un.org/sites/default/files/2020-06/Thailand-Migration-Report-2019.pdf.

[11] International Organization for Migration Migration Context 2021. https://thailand.iom.int/migration-context.

[12] Thepgumpanat P. Thai Lockdown Sparks Exodus of 60,000 Migrant Workers: Ministry Official. 2020. https://www.reuters.com/article/us-health-coronavirus-thailand-exodus-idUSKBN21C0ZI.

[13] Pross C. (2021). Migrant Workers in Times of COVID-19: An Empathetic Disaster Response for Myanmar Workers in Thailand. https://www.sei.org/perspectives/migrant-workers-covid-disaster-response/.

[14] Tangsathaporn P. (2021). Migrants Get Rough Deal under COVID. https://www.bangkokpost.com/thailand/general/2238135/migrants-get-rough-deal-under-covid.

[15] International Labour Organization (ILO) (2020). COVID-19: Impact on Migrant Workers and Country Response in Thailand. https://www.ilo.org/asia/publications/issue-briefs/WCMS_741920/lang--en/index.htm.

[16] Wiriyapong N. (2021). Don’t Leave Migrant Workers Behind. https://www.bangkokpost.com/business/2154731/dont-leave-migrant-workers-behind.

[17] Wipatayotin A. (2021). Delta Strain to Dominate in the Capital. https://www.bangkokpost.com/thailand/general/2140051/delta-strain-to-dominate-in-the-capital.

[18] Creswell J.W., Clark V.L.P. (2017). Designing and Conducting Mixed Methods Research.

[19] IOM (2020). Effects of COVID-19 on Migrants- Survey in Central America and Mexico (June 2020). https://dtm.iom.int/reports/effects-covid-19-migrants-survey-central-america-and-mexico-june-2020.

[20] Baloran E.T. (2020). Knowledge, attitudes, anxiety, and coping strategies of students during COVID-19 pandemic. J. Loss Trauma.

[21] Braun V., Clarke V. (2006). Using thematic analysis in psychology. Qual. Res. Psychol..

[22] Vergara M.B., Noom S.H. (2014). Acculturative stress and coping among Burmese women migrant workers in Thailand. AU J. Manag..

[23] Lam K.K., Johnston J.M. (2015). Depression and health-seeking behaviour among migrant workers in Shenzhen. Int. J. Soc. Psychiatry.

[24] Meyer S.R., Decker M.R., Tol W.A., Abshir N., Mar A.A., Robinson W.C. (2016). Workplace and security stressors and mental health among migrant workers on the Thailand–Myanmar border. Soc. Psychiatry Psychiatr. Epidemiol..

[25] Nadim W., AlOtaibi A., Al-Mohaimeed A., Ewid M., Sarhandi M., Saquib J., Alhumdi K., Alharbi A., Taskin A., Migdad M. (2016). Depression among migrant workers in Al-Qassim, Saudi Arabia. J. Affect. Disord..

[26] Khaled S.M., Gray R. (2019). Depression in migrant workers and nationals of Qatar: An exploratory cross-cultural study. Int. J. Soc. Psychiatry.

[27] Mucci N., Traversini V., Giorgi G., Tommasi E., De Sio S., Arcangeli G. (2019). Migrant workers and psychological health: A systematic review. Sustainability.

[28] Hasan S.I., Yee A., Rinaldi A., Azham A.A., Mohd Hairi F., Amer Nordin A.S. (2021). Prevalence of common mental health issues among migrant workers: A systematic review and meta-analysis. PLoS ONE.

[29] Htay M.N.N., Latt S.S., Maung K.S., Myint W.W., Moe S. (2020). Mental well-being and its associated factors among Myanmar migrant workers in Penang, Malaysia. Asia Pac. J. Public Health.

[30] Kumar K., Mehra A., Sahoo S., Nehra R., Grover S. (2020). The psychological impact of COVID-19 pandemic and lockdown on the migrant workers: A cross-sectional survey. Asian J. Psychiatry.

[31] Kesornsri S., Sitthimongkol Y., Punpuing S., Vongsirimas N., Hegadoren K.M. (2019). Mental health and related factors among migrants from Myanmar in Thailand. J. Popul. Soc. Stud. [JPSS].

[32] Guha P., Islam B., Hussain M.A. (2021). COVID-19 lockdown and penalty of joblessness on income and remittances: A study of inter-state migrant labourers from Assam, India. J. Public Aff..

[33] Chavan B., Sidana A., Arun P., Rohilla R., Singh G.P., Solanki R., Aneja J., Murara M.K., Verma M., Chakraborty S. (2021). Factors leading to reverse migration among migrant workers during the COVID-19 pandemic: A multicenter study from Northwest India. Prim. Care Companion CNS Disord..

[34] Wise J. (2021). COVID-19: Migrants face barriers accessing healthcare during the pandemic, report shows. Br. Med. J..

[35] Fu L., Lindenmeyer A., Phillimore J., Lessard-Phillips L. (2022). Vulnerable migrants’ access to healthcare in the early stages of the COVID-19 pandemic in the UK. Public Health.

[36] Knights F., Carter J., Deal A., Crawshaw A.F., Hayward S.E., Jones L., Hargreaves S. (2021). Impact of COVID-19 on migrants’ access to primary care and implications for vaccine roll-out: A national qualitative study. Br. J. Gen. Pract..

[37] Spiritus-Beerden E., Verelst A., Devlieger I., Langer Primdahl N., Botelho Guedes F., Chiarenza A., De Maesschalck S., Durbeej N., Garrido R., Gaspar de Matos M. (2021). Mental health of refugees and migrants during the COVID-19 pandemic: The role of experienced discrimination and daily stressors. Int. J. Environ. Res. Public Health.

[38] UCANews (2022). Thai Authorities Deport Desperate Migrants from Myanmar. https://www.ucanews.com/news/thai-authorities-deport-desperate-migrants-from-myanmar/96852.

[39] Irrawaddy T. (2022). Thai Prisons Crowded with Illegal Myanmar Migrants. https://www.irrawaddy.com/news/burma/thai-prisons-crowded-with-illegal-myanmar-migrants.html.

[40] Weller S.J., Crosby L.J., Turnbull E.R., Burns R., Miller A., Jones L., Aldridge R.W. (2019). The negative health effects of hostile environment policies on migrants: A cross-sectional service evaluation of humanitarian healthcare provision in the UK. Wellcome Open Res..

[41] IOM (2021). Despite Positive Efforts, Too Many Migrants Face Challenges Accessing COVID-19 Vaccines. https://www.iom.int/news/despite-positive-efforts-too-many-migrants-face-challenges-accessing-covid-19-vaccines.

[42] Desie Y., Habtamu K., Asnake M., Gina E., Mequanint T. (2021). Coping strategies among Ethiopian migrant returnees who were in quarantine in the time of COVID-19: A center-based cross-sectional study. BMC Psychol..

[43] Berry J.W. (1997). Immigration, acculturation, and adaptation. Appl. Psychol..

[44] Du H., Li X. (2015). Acculturation and HIV-related sexual behaviours among international migrants: A systematic review and meta-analysis. Health Psychol. Rev..

[45] Kuo B.C. (2014). Coping, acculturation, and psychological adaptation among migrants: A theoretical and empirical review and synthesis of the literature. Health Psychol. Behav. Med.: Open Access J..

[46] Fancourt D., Finn S. (2019). What Is the Evidence on the Role of the Arts in Improving Health and Well-Being? A Scoping Review.

[47] Johannes N., Vuorre M., Przybylski A.K. (2021). Video game play is positively correlated with well-being. R. Soc. Open Sci..

[48] Melodia F., Canale N., Griffiths M.D. (2020). The role of avoidance coping and escape motives in problematic online gaming: A systematic literature review. Int. J. Ment. Health Addict..

[49] Rogers R.W. (1975). A protection motivation theory of fear appeals and attitude change1. J. Psychol..

[50] Westcott R., Ronan K., Bambrick H., Taylor M. (2017). Expanding protection motivation theory: Investigating an application to animal owners and emergency responders in bushfire emergencies. BMC Psychol..

[51] Floyd D.L., Prentice-Dunn S., Rogers R.W. (2000). A meta-analysis of research on protection motivation theory. J. Appl. Soc. Psychol..

[52] Weishaar H.B. (2008). Consequences of international migration: A qualitative study on stress among Polish migrant workers in Scotland. Public Health.

[53] Weishaar H.B. (2010). “You have to be flexible”—Coping among polish migrant workers in Scotland. Health Place.

[54] Tang S., Li X. (2021). Responding to the pandemic as a family unit: Social impacts of COVID-19 on rural migrants in China and their coping strategies. Humanit. Soc. Sci. Commun..

[55] Srivastava A., Arya Y.K., Joshi S., Singh T., Kaur H., Chauhan H., Das A. (2021). Major stressors and coping strategies of internal migrant workers during the COVID-19 pandemic: A qualitative exploration. Front. Psychol..

[56] Magis K. (2010). Community resilience: An indicator of social sustainability. Soc. Nat. Resour..

[57] Wang J.-L., Zhang D.-J., Zimmerman M.A. (2015). Resilience theory and its implications for Chinese adolescents. Psychol. Rep..

[58] Folkman S. (1984). Personal control and stress and coping processes: A theoretical analysis. J. Personal. Soc. Psychol..

[59] Lazarus R.S., Folkman S. (1984). Stress, Appraisal, and Coping.

[60] Olukotun O., Gondwe K., Mkandawire-Valhmu L. (2019). The mental health implications of living in the shadows: The lived experience and coping strategies of undocumented African migrant women. Behav. Sci..

[61] Nakonz J., Shik A.W.Y. (2009). And all your problems are gone: Religious coping strategies among Philippine migrant workers in Hong Kong. Ment. Health Relig. Cult..

[62] Tschirhart N., Straiton M., Ottersen T., Winkler A.S. (2019). “Living like I am in Thailand”: Stress and coping strategies among Thai migrant masseuses in Oslo, Norway. BMC Women’s Health.

[63] Dull V.T., Skokan L.A. (1995). A cognitive model of religion’s influence on health. J. Soc. Issues.

[64] Ang J., Chia C., Koh C., Chua B., Narayanaswamy S., Wijaya L. (2017). Healthcare-seeking behaviour, barriers and mental health of non-domestic migrant workers in Singapore. BMJ Glob. Health.

[65] Slootjes J., Keuzenkamp S., Saharso S. (2018). Narratives of meaningful endurance–how migrant women escape the vicious cycle between health problems and unemployment. Comp. Migr. Stud..

